# GPR55 Antagonist CID16020046 Suppresses Collagen-Induced Rheumatoid Arthritis by Suppressing Th1/Th17 Cells in Mice

**DOI:** 10.3390/ijms26104680

**Published:** 2025-05-14

**Authors:** Jung-Eun Lee, Dong-Soon Im

**Affiliations:** 1 Department of Biomedical and Pharmaceutical Sciences, Graduate School, Kyung Hee University, Seoul 02446, Republic of Korea; xkdnj1005@khu.ac.kr; 2 Department of Basic Pharmaceutical Sciences, Graduate School, Kyung Hee University, Seoul 02446, Republic of Korea

**Keywords:** G-protein-coupled receptor 55, GPR55, rheumatoid, arthritis, CID16020046

## Abstract

Lysophosphatidylinositols are degradation products of phosphatidylinositols within cell membranes and digestive metabolites of a high-fat diet in the gut. G-protein-coupled receptor 55 (GPR55) is a receptor that senses lysophosphatidylinositol and acts as an immune mediator, being primarily upregulated during immune cell activation. This study aimed to investigate the role of GPR55, using its antagonist, CID16020046, in a collagen-induced rheumatoid arthritis mouse model. It was observed that DBA-1J mice develop joint lesions characteristic of rheumatoid arthritis following immunization with bovine type II collagen. The administration of CID16020046 (1 mg/kg, intraperitoneally) alleviated rheumatoid arthritis symptoms and inflammatory responses. Histopathological analysis showed that CID16020046 reduced foot edema, proteoglycan loss, and bone erosion in the joints. CID16020046 also decreased rheumatoid-arthritis-induced serum IgG levels, as measured using enzyme-linked immunosorbent assays. The treatment reduced levels of pro-inflammatory cytokines (IL-1β and IL-6), Th1 cytokine (IFN-γ), and Th17 cytokine (IL-17A), along with matrix metalloproteinase-3 (MMP-3) and the receptor activator of nuclear factor-κB ligand (RANKL) in the feet. A significant reduction in splenomegaly was also observed, along with significant reductions in CD4^+^ T helper 1 (Th1) and Th17 cells in the spleen. Additionally, CID16020046 suppressed the differentiation of naïve T cells into CD4^+^IL-17^+^ Th17 cells. CID16020046 suppressed expression levels of inflammatory cytokine mRNAs in SW982 human synovial cells. In conclusion, blocking GPR55 alleviates collagen-induced rheumatoid arthritis symptoms by suppressing Th1 and Th17 cells in the spleen and pro-inflammatory cytokines in the joints, suggesting that GPR55 is a potential therapeutic target for autoimmune inflammatory diseases.

## 1. Introduction

Lysophosphatidylinositols (LPIs) are degradation products of phosphatidylinositols within cell membranes [1] and digestive metabolites of a high-fat diet in the gut [2]. Circulating levels of LPI have been found to increase in obese patients [3], and LPI (18:2) levels in serum are elevated in patients with rheumatoid arthritis (RA) compared to controls [4]. LPIs are endogenous ligands for the G-protein-coupled receptor 55 (GPR55) [5,6]. GPR55 expression has been observed in synoviocytes, osteoclasts, osteoblasts, chondrocytes, and various immune system components, including macrophages, T cells, and microglia [7,8,9,10,11,12,13,14,15]. In a rat model of acute joint inflammation, the administration of O-1602, a GPR55 agonist, reduced nociception in a GPR55-dependent manner [16]. The role of GPR55 in regulating osteoclast numbers and bone turnover has been studied in vivo [12]. Cannabidiol, a GPR55 antagonist, inhibited bone resorption in vivo by modulating GPR55 signaling [17]. GPR55 activation in chondrocytes induces the production of matrix metalloproteinases [12].

LPIs play a crucial role in modulating inflammation by activating GPR55 [1]. CID16020046, a specific GPR55 antagonist, reduced colon inflammation in a murine colitis model [9]. Although the anti-inflammatory effects of CID16020046 have been studied in conditions such as atherosclerosis, colitis, hepatic steatosis, and obese asthma [9,18,19,20,21], its role in rheumatoid arthritis has not yet been explored. Therefore, this study utilized CID16020046 to investigate the role of GPR55 in a murine collagen-induced rheumatoid arthritis model.

## 2. Results

### 2.1. CID16020046 Treatment Inhibited the Progression of Arthritis and the Thickening of the Feet in DBA-1J Mice

**Figure 1:**
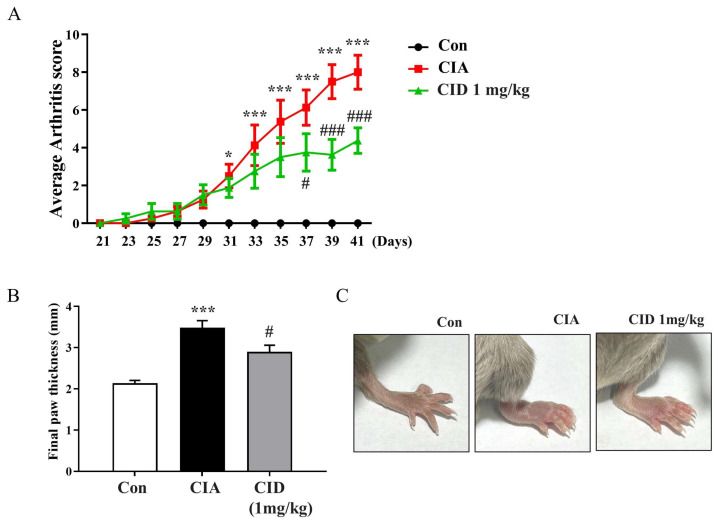
CID1602246 reduced the CIA-induced increase in arthritis score and paw thickness in DBA-1J mice. (**A**) Arthritis scores from day 21 to day 41 in DBA-1J mice. (**B**) Final paw thickness on day 42 in DBA-1J mice. (**C**) Representative foot images from DBA-1J mice. Data are presented as mean ± SEM (*n* = 8). *** *p* < 0.001, * *p* < 0.05, compared to the control group; ### *p* < 0.001, # *p* < 0.05, compared to the CIA group in DBA-1J mice.

To investigate the therapeutic potential of GPR55 in CIA, macroscopic clinical features were evaluated starting on the 21st day after incomplete Freund’s adjuvant (IFA) injection in the DBA-1J mice (Figure 1A). From day 31, the arthritis score, reflecting paw swelling, significantly increased, peaking at around day 41. The administration of CID16020046 led to a marked reduction in the arthritis score from day 37 onwards compared to that of the CIA group, indicating a significant decrease in foot edema (Figure 1A). On day 42, the final paw thickness was measured prior to sacrifice (Figure 1B). In the CIA group, the paw thickness was notably greater than that observed in the control group, while CID16020046 administration significantly reduced the CIA-induced increase in foot thickness (Figure 1A,B). Figure 1C shows the visibly swollen feet observed in the CIA group and the reduction in swelling in the CID16020046-treated mice. Thus, CID16020046 suppresses the CIA-induced increase in arthritis score and foot thickening by inactivating GPR55.

### 2.2. CID16020046 Treatment Reduced the Histological Alterations Associated with Rheumatoid Arthritis in DBA-1J Mice

**Figure 2:**
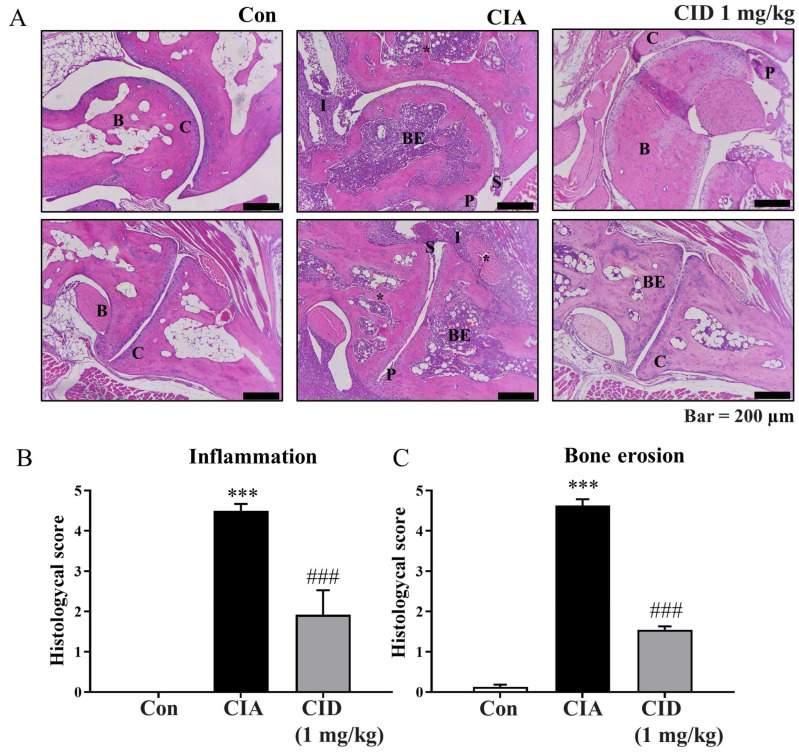
CID16020046 diminished CIA-induced inflammation and bone erosion in DBA-1J mice. (**A**) H&E staining (100×) of the ankle joint in DBA-1J mice. (**B**,**C**) Histologic scores based on (**B**) inflammation and (**C**) bone erosion in DBA-1J mice. Data are shown as mean ± SEM (*n* = 8). *** *p* < 0.001, compared to the control group; ### *p* < 0.001, compared to the CIA group in DBA-1J mice. B, bone; BE, bone erosion; C, cartilage damage; I, inflammation; P, pannus tissue formation; S, synovial hyperplasia; *, trabecular bone loss.

Histological examinations using H&E staining were performed to assess inflammation and bone erosion. Synovial hyperplasia, a thickening of the synovium typical in rheumatoid arthritis, was observed in the CIA group of DBA-1J mice (Figure 2A). As the synovial tissue enlarges, it forms a ‘pannus’ structure, which damages the cartilage and bones surrounding the joint. Pannus formation was more pronounced in the CIA group (Figure 2A). However, treatment with CID16020046 reduced both synovial hyperplasia and pannus formation in these mice (Figure 2A). Inflammation levels were quantified and are represented as histograms (Figure 2B). CIA significantly increased inflammation scores, but CID16020046 treatment reduced this increase in the mice (Figure 2B). Bone erosion levels were also quantified and are displayed as histograms (Figure 2C), with CIA causing a significant rise in bone erosion scores, which were lessened by CID16020046 in the mice (Figure 2C). These results suggest that CID16020046 alleviates CIA-induced histological changes by inactivating GPR55.

**Figure 3:**
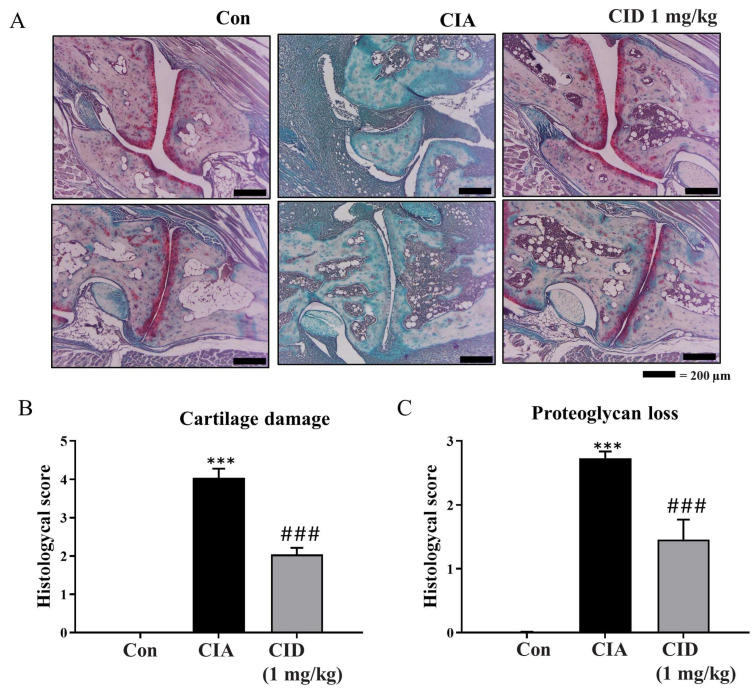
CID16020046 diminished CIA-induced cartilage damage and proteoglycan loss in DBA-1J mice. (**A**) Safranin-O (100x) of the ankle joint in DBA-1J mice. (**B**,**C**) Histologic scores based on (**B**) cartilage damage and (**C**) proteoglycan loss in DBA-1J mice. Data are shown as mean ± SEM (*n* = 8). *** *p* < 0.001, compared to the control group; ### *p* < 0.001, compared to the CIA group in DBA-1J mice.

Moreover, Safranin-O staining was employed to evaluate cartilage damage and proteoglycan loss. Proteoglycan levels, a vital component of the articular cartilage extracellular matrix, were indicated with red staining (Figure 3A). As shown in Figure 3A, cartilage damage was more extensive in the CIA group compared to that of the controls, but treatment with CID16020046 partially mitigated this damage. The degree of cartilage damage was evaluated and is depicted as histograms in Figure 3B. While CIA significantly increased cartilage damage, CID16020046 reduced this effect in the DBA-1J mice (Figure 3B). Compared to the control group, proteoglycan loss was significant in the CIA group; however, CID16020046 administration provided partial protection against proteoglycan loss in the mice (Figure 3A,C). These results suggest that CID16020046 offers protection against CIA-induced cartilage damage and proteoglycan loss by inactivating GPR55.

### 2.3. CID16020046 Treatment Reduced mRNA Expression Levels of Pro-Inflammatory Cytokines in Foot Tissues in DBA-1J Mice

**Figure 4:**
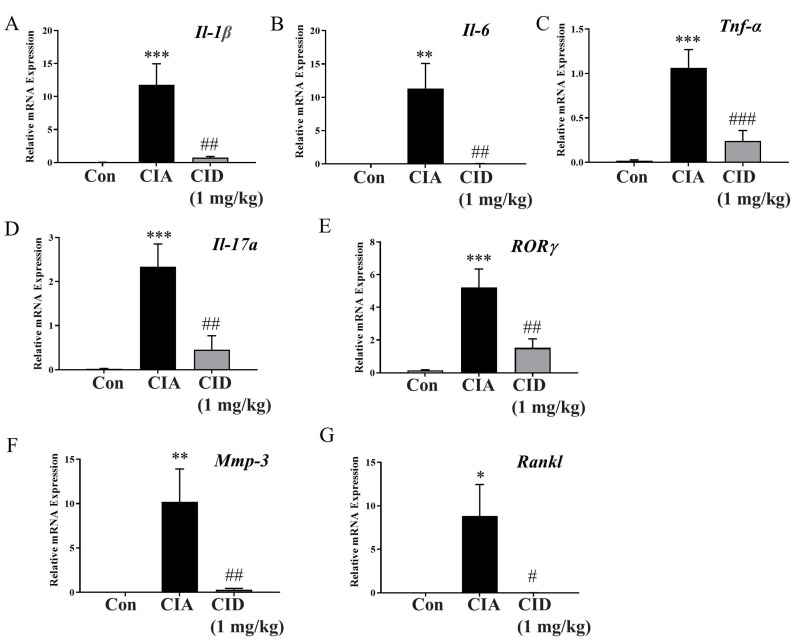
CID16020046 reduced the CIA-induced elevation of inflammatory cytokine levels in the feet of DBA-1J mice. Cytokine mRNA levels were normalized to the Gapdh mRNA level. Data are shown as mean ± SEM (*n* = 8). (**A**) *Il-1β*, (**B**) *Il-6*, (**C**) *Tnf-α*, (**D**) *Il-17a*, (**E**) *ROR-γ*, (**F**) *Mmp-3*, and (**G**) *Rankl* in DBA-1J mice. Significance levels: *** *p* < 0.001, ** *p* < 0.01, * *p* < 0.05, compared to the control group; ### *p* < 0.001, ## *p* < 0.01, # *p* < 0.05, compared to the CIA group in DBA-1J mice.

As rheumatoid arthritis is a systemic autoimmune disease, changes in mRNA expression levels of pro-inflammatory cytokines were examined in foot tissues collected on the 42nd day. In the CIA group, the mRNA levels of pro-inflammatory cytokines (Il-1β, Il-6, and Tnf-α) and the Th17 cytokine (Il-17a) were higher than those observed in the control group (Figure 4A–D). However, treatment with CID16020046 lowered these cytokine levels in the mice (Figure 4A–D). Additionally, mRNA expression levels of RORγ, a key transcription factor for Th17 cells, were increased in the CIA group compared to those of the controls (Figure 4E), but CID16020046 reduced its expression levels (Figure 4E). Furthermore, in the CIA group, mRNA levels of matrix metalloproteinase-3 (Mmp-3), a protease involved in joint destruction, and the receptor activator of nuclear factor-κB ligand (Rankl), which promotes osteoclast activity for bone resorption, were elevated (Figure 4F,G). Therefore, CID16020046 significantly diminished these increases in the mice (Figure 4F,G).

### 2.4. CID16020046 Treatment Suppressed Serum IgG Levels in DBA-1J Mice

**Figure 5:**
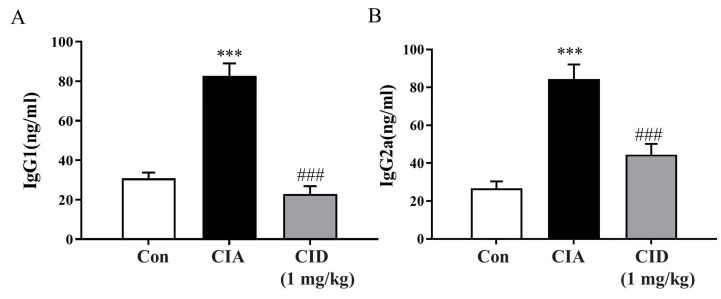
CID16020046 reduced CIA-induced increases in serum IgG1 and IgG2a levels in DBA-1J mice. Blood samples were collected on day 42. Serum levels of IgG1 (**A**) and IgG2a (**B**) were determined by ELISA in DBA-1J mice. Data are expressed as mean ± SEM (*n* = 8). *** *p* < 0.001, compared to the control group; ### *p* < 0.001, compared to the CIA group in DBA-1J mice.

Rheumatoid arthritis is characterized by an immune response driven by autoantibodies that erroneously target and interact with joint antigens. IgG, the most common autoantibody, is frequently elevated in rheumatoid arthritis. Different IgG subclasses play roles in immune functions and phagocytosis, contributing to inflammation. The IgG1 and IgG2a subclasses, important for complement activation, were measured. In the CIA group, the levels of both IgG1 and IgG2a were significantly elevated compared to those of the control group. However, treatment with CID16020046 effectively reduced these levels in the mice (Figure 5A,B).

### 2.5. CID16020046 Treatment Reduced Spleen Enlargement in DBA-1J Mice

**Figure 6:**
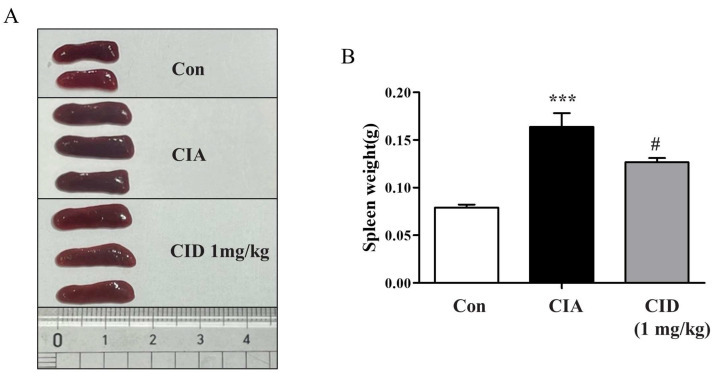
CID16020046 reduced CIA-induced spleen enlargement in DBA-1J mice. (**A**) Images of spleens. (**B**) Spleen weights in DBA-1J mice. The results are shown as mean ± SEM (*n* = 8). *** *p* < 0.001, compared to the control group; # *p* < 0.05, compared to the CIA group in DBA-1J mice.

The spleen, an essential organ within the immune system, was weighed to evaluate any changes. In mice from the CIA group, there was a notable increase in spleen weight compared to that of the control group (Figure 6A,B). However, administering CID16020046 significantly reduced the gain in spleen weight in these mice (Figure 6A,B).

### 2.6. CID16020046 Treatment Diminished the CIA-Induced Rise in Populations of Th1 and Th17 Cells in the Spleens of DBA-1J Mice

**Figure 7:**
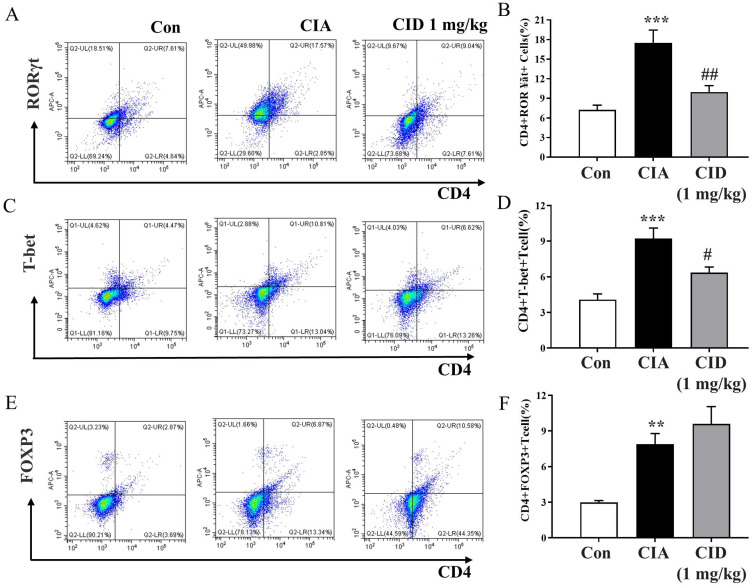
CID16020046 reduced CIA-induced increases in the populations of Th1 and Th17 cells in DBA-1J mice. Representative plot images for CD4^+^RORγt^+^ Th17 cells (**A**), CD4^+^T-bet^+^ Th1 cells (**C**), and CD4^+^FoxP3^+^ Treg cells (**E**). Percentages of CD4^+^RORγt^+^ Th17 cells (**B**), CD4^+^T-bet^+^ Th1 cells (**D**), and CD4^+^FoxP3^+^ Treg cells (**F**) in DBA-1J mice. The results are shown as mean ± SEM (*n* = 8). *** *p* < 0.001, ** *p* < 0.01, compared to the control group; ## *p* < 0.01, # *p* < 0.05, compared to the CIA group in DBA-1J mice.

The imbalance between RORγt^+^ type 17 helper T cells (Th17) and FoxP3^+^ regulatory T cells (Treg) in synovial lesions plays a crucial role in the development of rheumatoid arthritis [18,19]. To evaluate the populations of Th1, Th17, and Treg cells in the spleen, FACS analysis was performed (Supplemental Figure S1). The induction of CIA resulted in a significant rise in the populations of CD4^+^T-bet^+^ Th1 cells and CD4^+^RORγt^+^ Th17 cells, but treatment with CID16020046 reduced the increase in these pro-inflammatory Th1 and Th17 cells in the mice (Figure 7A–D). Although CIA induction also led to a significant increase in the populations of anti-inflammatory CD4^+^FoxP3^+^ Treg cells, CID16020046 only slightly enhanced Treg cell populations, without significant impact (Figure 7E,F).

### 2.7. CID16020046 Treatment Inhibited the Differentiation of Naïve T Cells into Th17 Cells

**Figure 8:**
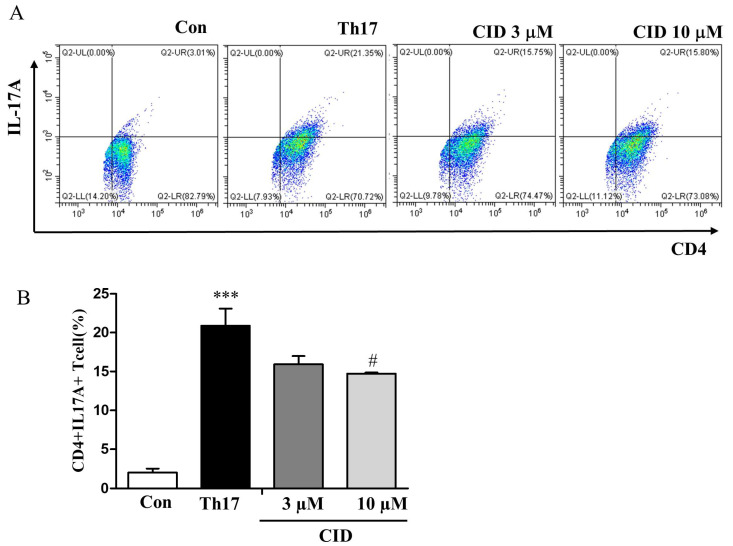
The suppressive effect of CID16020046 on T cell differentiation into Th17 cells. CD4^+^ T cells, isolated from splenocytes, were cultured in media for Th17 cell differentiation for 5 days in plates pre-coated with an antibody to mouse CD3. (**A**) Representative plot images. (**B**) Histograms show the percentage of CD4^+^IL-17A^+^ cells (**B**) (*n* = 5). *** *p* < 0.001, compared to the control group; # *p* < 0.05, compared to the Th17 differentiation group.

Rheumatoid arthritis is primarily driven by Th17 cells, which are pivotal in its development. Observing notably high Th17a cytokine levels and an increased population of CD4^+^RORγt^+^ Th17 cells in the foot and spleen, we explored the impact of CID16020046 on the differentiation of splenic CD4^+^ naïve T cells into Th17 cells. After culturing these naïve T cells in Th17-specific differentiation media and performing FACS analysis, we observed Th17 differentiation marked by a rise in the CD4^+^IL-17A^+^ T cell population, as shown in Figure 8A,B. Treatment with CID16020046 reduced Th17 cell differentiation in a concentration-dependent manner (Figure 8A,B). These findings indicate that administering CID16020046 might impede the transformation of naïve T cells into inflammatory Th17 cells, thereby decreasing inflammatory cytokine production and alleviating arthritis symptoms.

### 2.8. CID16020046 Treatment Reduced the mRNA Expression Levels of Inflammatory Cytokines in SW982 Human Synovial Cells

**Figure 9:**
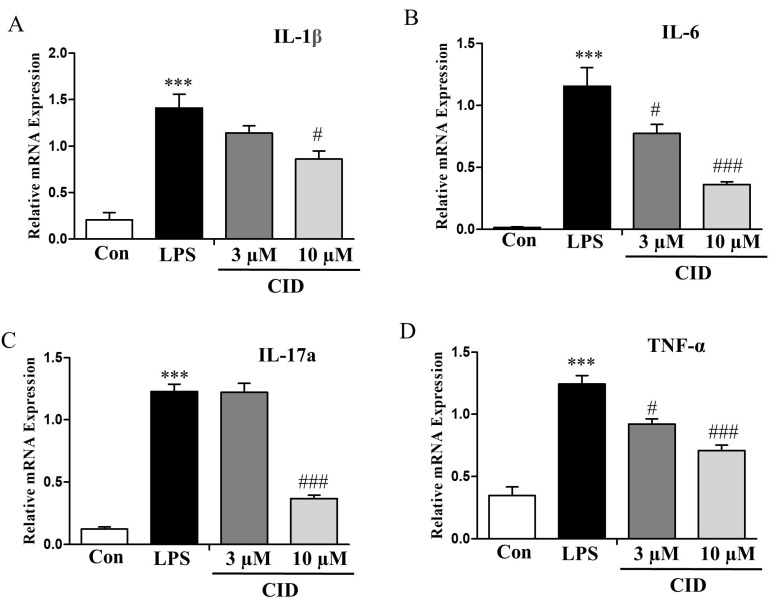
CID16020046 inhibited LPS-induced increase in mRNA expression levels of pro-inflammatory cytokines in SW982 cells. SW982 cells were seeded at 1 × 10^5^/mL. After 24 h, CID16020046 was treated at the concentration indicated, and then LPS (100 ng/mL) was added and incubated for 30 min. qPCR was used to confirm the mRNA expression levels of inflammatory cytokines in SW982 cells. (**A**) IL-1β, (**B**) IL-6, (**C**) IL-17A, and (**D**) TNF-α. The results are presented as mean ± SEM (*n* = 5). *** *p* < 0.001, vs. the control group; ### *p* < 0.001, # *p* < 0.05, vs. the LPS-induced group.

The synovial membrane, which becomes hyperplastic in rheumatoid arthritis, produces cytokines that contribute to cartilage deterioration and are likely involved in the disease’s chronic progression. To determine if CID16020046 can influence inflammatory responses in this membrane, researchers used the human synovial cell line SW982. The mRNA levels of inflammatory cytokines in SW982 cells stimulated with lipopolysaccharide (LPS) were quantified using qRT-PCR. LPS treatment elevated the expression of multiple inflammatory cytokines (IL-1β, IL-6, IL-17a, and TNF-α), but the presence of CID16020046 significantly decreased these levels in a dose-dependent fashion (Figure 9A–D).

## 3. Discussion

The expression of GPR55 has been identified in osteoclasts and osteoblasts of both humans and mice [12], human chondrocytes [11,15], as well as in canine T cells, neutrophils, and synoviocytes [10,14]. This suggests that GPR55 might be involved in the regulation of synovial inflammation and joint damage associated with rheumatoid arthritis and osteoarthritis [14]. In our current research, we present novel evidence that the GPR55 antagonist CID16020046 can inhibit lipopolysaccharide-induced expression of pro-inflammatory cytokines in SW982 human synoviocytes and mitigate collagen-induced rheumatoid arthritis in DBA-1J mice. The ability of CID16020046 to reduce proteoglycan depletion and bone erosion verifies its suppressive impact on joint inflammation. A dosage of 1 mg/kg of CID16020046, previously effective in various murine models like atherosclerosis, atopic dermatitis, hepatic steatosis, and obesity-related asthma [2,20,21,22], was utilized in our study. The reduction in serum IgG levels and spleen weights due to CID16020046 administration provides initial insights into its mechanism of action, specifically through immune suppression. Furthermore, the modulation of mRNA expression levels of inflammatory cytokines in the joints by CID16020046 strongly supports its anti-inflammatory and immune suppressive effects, aligning with observations in an obese asthma model [21]. Additionally, the ability of CID16020046 to decrease the populations of pro-inflammatory Th1 and Th17 cells reinforces its role in immune suppression. These alterations in Th1/Th17 cell populations correspond well with changes in cytokine levels associated with Th1 (*Ifn-γ*) and Th17 (*Il-17a*) cells [21,22]. Moreover, the downregulation of *Mmp-3* and *Rankl* expression levels by CID16020046 offers tangible evidence of its actions within the joints. Lastly, the inhibition of Th17 cell differentiation by CID16020046 suggests its primary site of action, as this leads to a decrease in Th17 cell numbers and their cytokine levels.

In light of GPR55’s role in promoting inflammation in various experimental models, such as colitis, non-alcoholic steatohepatitis, atherosclerosis, and asthma associated with obesity [1,2,9,20,21,23], this research explores the potential of inhibiting GPR55 for treating autoimmune diseases affecting the joints. The reduction in rheumatoid arthritis symptoms by CID16020046 seems to stem from its ability to inhibit Th1 and Th17 cells, as its effect on immune responses was not observed in Treg cells within the spleen. These findings underscore the pro-inflammatory role of GPR55, suggesting that its antagonism through CID16020046 may be beneficial in treating rheumatoid arthritis. Although we detected Th1, Th17, and Treg cells in the splenocytes by labeling both CD4 and RORγt, FoxP3, or T-bet, confirmative gating strategy to exclude CD4^+^ monocytes or macrophages was not applied, limiting the interpretation of the results. Given the elevated levels of endogenous ligands, LPIs, in the blood of rheumatoid arthritis patients [4], it is plausible that the activation of GPR55 by LPIs contributes to inflammatory and autoimmune processes, as demonstrated through the use of the GPR55 antagonist CID16020046 in this study.

On a mechanistic level, GPR55 antagonists such as KIT 17 and ML193 have been shown to markedly inhibit the release of prostaglandin E_2_ in lipopolysaccharide-stimulated primary microglia [24]. Additionally, the antagonist CID16020046 significantly decreases the production of pro-inflammatory cytokines TNF-α and IL-6 and reduces leukocyte adherence in the submucosal venules of the intestines during experimental sepsis [25]. Thus, GPR55’s involvement in inflammatory immune responses extends beyond Th1/Th17 cells to include synoviocytes, chondrocytes, osteoclasts, microglia, and endothelial cells [10,11,12,14,15]. Although human SW982 synovial cells were studied, extensive studies on GPR55 in chondrocytes, osteoclasts, macrophages, and neutrophils are necessary to elucidate the mode of action of CID16020046 in rheumatoid arthritis. We anticipate that the utilization of GPR55-gene-deficient mice may provide insights into its functions in autoimmune diseases and its therapeutic potential. More preclinical studies with CID16020046 would accelerate the development of GPR55-targeting drugs, although human dosage, efficacy, and toxicity must be primarily overcome.

## 4. Materials and Methods

### 4.1. Materials

CID16020046 (4-[4,6-dihydro-4-(3-hydroxyphenyl)-3-(4-methylphenyl)-6-oxopyrrolo [3,4-c]pyrazol-5(1H)-yl]-benzoic acid, Cat no. 834903-43-4, PubChem Substance ID: 16020046, Molecular Weight: 425.4, Purity: ≥98% in HPLC) was purchased from Cayman Chemical (Ann Arbor, MI, USA). Bovine type II collagen, complete Freund’s adjuvant (CFA), and incomplete Freund’s adjuvant (IFA) were acquired from Chondrex Inc. in Woodinville, WA, USA. Other materials were purchased from Sigma-Aldrich (St. Louis, MO, USA).

### 4.2. Cell Culture

Human SW982 synovial cells were obtained from the American Type Culture Collection (ATCC) in Manassas, VA, USA. The cells were cultured in Dulbecco’s Modified Eagle Medium (DMEM) supplemented with 10% (*v*/*v*) heat-inactivated fetal bovine serum, 100 U/mL penicillin, and 50 μg/mL streptomycin. They were maintained at 37 °C in a humidified incubator with 5% CO_2_.

### 4.3. Animals

We obtained male DBA-1J mice aged 7 weeks old from Orient Bio Co., Ltd. (Seoul, Republic of Korea). The mice were housed three per cage in standard plastic cages with sawdust bedding. They were provided with standard laboratory chow and water ad libitum. A 12 h light–dark cycle was maintained at 22 °C and 60% humidity. The experimental protocols followed the rules and regulations of the Animal Ethics Committee of Kyung Hee University (KHSASP-24-527).

### 4.4. Human SW982 Synovial Cell Treatment

For experimental preparation, SW982 cells were seeded at a density of 1 × 10^5^ cells per well in six-well plates and cultured overnight. The next day, the medium was removed, and the wells were rinsed with PBS. The cells were then treated with CID16020046 and incubated for 30 min. Subsequently, lipopolysaccharide (LPS; 100 ng/mL) was added, and the cells were cultured overnight before being collected for qRT-PCR analysis.

### 4.5. Induction of Rheumatoid Arthritis in DBA-1J Mice and CID16020046 Administration

CIA is a widely used autoimmune animal model for studying rheumatoid arthritis. Male DBA-1J mice, aged 7 to 9 weeks old, and weighing 23 to 25 g, were randomly assigned to three experimental groups (*n* = 8 per group), as follows: the control group, CIA group, and CIA + CID16020046-treated group. A complete Freund’s adjuvant (CFA) emulsion was prepared as previously described, and 100 μL was injected into the tails of anesthetized mice with Avertin on day 0 (D0). A booster injection of incomplete Freund’s adjuvant (IFA) was administered on day 21. CID16020046 (1 mg/kg body weight) was given by intraperitoneal injection 30 min before the emulsion injection. The CID16020046 treatment began on day 21 and continued until day 41, during which the clinical scores and body weights of the mice were evaluated.

### 4.6. Measurement of the Severity of Arthritis

Starting on day 21, the severity of arthritis in the mice was assessed every other day using a scoring system based on previous studies. The scoring criteria were as follows: 0 indicated no signs of arthritis; 1 represented swelling and/or redness of the paw or a single digit; 2 indicated involvement of two joints; 3 signified involvement of more than two joints; and 4 denoted severe arthritis affecting the entire paw and digits. An arthritis index score for each mouse was calculated by summing the scores from each individual paw [22,25,26].

### 4.7. Histological Assessment of Arthritis

The Korea non-clinical technology solution center (Seoul, Republic of Korea) prepared paraffin sections from the tissues of DBA-1J mice feet. These sections were used to identify histological changes related to rheumatoid arthritis through Safranin-O staining, utilizing the NovaUltra Safranin-O Stain Kit (cat. IW-3011, IHC WORLD, Ellicott City, MD, USA), following the manufacturer’s instructions. For hematoxylin and eosin (H&E) staining, the paraffin-embedded sections were first deparaffinized by immersion in xylene for 5 min, followed by dehydration with ethanol. After treatment with hematoxylin, the sections were rinsed with tap water, re-dehydrated with ethanol, stained with eosin Y, fixed with ethanol, and finally mounted for observation using Permount. Inflammation, pannus formation, and erosion of cartilage and bone were assessed using previously established indices [27]. Inflammation and bone erosion were rated on a scale from 0 to 5, as follows: 0 indicated normal; 1 for minimal changes (local inflammatory cell infiltration or mild swelling); 2 for mild changes (local inflammatory cell infiltration and mild swelling); 3 for moderate resorption of trabecular and cortical bone without defects or loss; 4 for marked changes (significant inflammatory cell infiltration and damage to cortical and trabecular bones); and 5 for severe infiltration of inflammatory cells and complete skeletal destruction. Cartilage damage was evaluated based on the loss of Safranin-O staining, scored on a semi-quantitative scale from 0 to 4, as follows: 0 for intact cartilage; 1 for minor damage; 2 for moderate damage; 3 for high damage; and 4 for severe damage [28,29].

### 4.8. Flowcytometric Analysis

To evaluate the T cell population, single cells isolated from spleens were stained with an FITC-conjugated rat antibody against CD4 (catalog no. 11-0041-82, eBioscience, San Diego, CA, USA) at 4 °C for 20 min. The cells were then fixed at room temperature for one hour using an intracellular fixation buffer (catalog no. 00-8222-49, eBioscience). After fixation, the cells were permeabilized with a permeabilization buffer (catalog no. 88-8824-00, eBioscience) and stained for one hour at room temperature with either APC-conjugated rat anti-FoxP3 (catalog no. 17-5773-82, eBioscience), eFluor 450-conjugated rat anti-T-bet (catalog no. 48-5825-82, eBioscience), or APC-conjugated rat anti-RORγt (catalog no. 17-6988-82). The analysis was performed using a CytoFLEX Flow cytometer (Beckman Coulter, Brea, CA, USA).

### 4.9. Quantitative Real-Time PCR

To assess the expression of inflammatory markers in the feet of mice by qRT-PCR, first-strand cDNA was synthesized from total RNA isolated using a TRIzol reagent (Invitrogen, Waltham, MA, USA); moreover, total RNA was isolated from the feet or SW982 human synovial cells. The RNA was transcribed reversely into cDNA using MMLV reverse transcriptase (Promega, Madison, WI, USA). Thunderbird Next SYBR qPCR Mix (Toyobo, Osaka, Japan) was used for qRT-PCR in a CFX Connect Real-Time System (Bio-Rad, Hercules, CA, USA). The following specific primers of *Mus musculus* were used: *Il-1β* (sense 5′-CAG GCA GGC AGT ATC ACT CA-3′, antisense 5′-TGT CCT CAT CCT GGA AGG TC-3′), *Tnf-α* (sense 5′-ACG GCA TGG ATC TCA AAG AC-3′, antisense 5′-GTG GGT GAG GAG CAC GTA GT-3′), *Il-6* (sense 5′-CTG ATG CTG GTG ACA ACC AC-3′, antisense 5′-TCC ACG ATT ACC CAG AGA AC-3′), *Il-17a* (sense 5′-CAGCGTGTCCAAACACTGAG-3′, antisense 5′-CGGTTGAGGTAGTCTGAGGG-3′), *Mmp-3* (sense 5′-CAG GTG TGG TGT TCC TGA TG-3′, antisense 5′-TTT CAA TGG CAG AAT CCA CA-3′), *Rankl* (sense 5′-GCA GAA GGA ACT GCA ACA CA-3′, antisense 5′-GAT GGT GAG GTG TGC AAA TG-3′), *RORγ* (sense 5′-TCC CGA GAT GCT GTC AAG TT-3′, antisense 5′-ACT TGT TCC TGT TGC TGC TG-3′), and *Gapdh* (sense 5′-AAC TTT GGC ATT GTG GAA GG-3′, antisense 5′-GGA TGC AGG GAT GAT GTT CT-3′); and for Homo sapiens: *GAPDH* (sense 5′-CCA CCC AGA AGA CTG TGG AT-3′ antisense 5′-TTC AGC TCA GGG ATG ACC TT-3′), *IL-1β* (sense 5′-GGA CAA GCT GAG GAA GAT GC-3′ antisense 5′-TCG TTA TCC CAT GTG TCG AA-3′), *IL-6* (sense 5′-GAA AGC AGC AAA GAG GCA CT-3′ antisense 5′-TTT CAC CAG GCA AGT CTC CT-3′), *IL-17A* (sense 5′-ATG AAC TCT GTC CCC ATC CA-3′ antisense 5′-CCC ACG GAC ACC AGT ATC TT-3′), and *TNF-α* (sense 5′-AAC CTC CTC TCT GCC ATC AA-3′ antisense 5′-GGA AGA CCC CTC CCA GAT AG-3′). Thermal cycling conditions were as follows: 1 cycle at 95 °C for 4 min; 40 cycles at 95 °C for 30 s and at 57 °C for 30 s; and 1 cycle at 95 °C for 30 s. The 2^−ΔΔCt^ method was used to calculate each gene expression.

### 4.10. Enzyme-Linked Immunosorbent Assay (ELISA)

Mouse sera were stored at –80 °C until analysis. The serum concentrations of IgG1 and IgG2a were measured using specific ELISA kits (eBioscience, San Diego, CA, USA; IgG1: catalog no. 88-50410-88, IgG2a: catalog no. 88-50420-88). Avidin–horseradish peroxidase was used for detection, and absorbance was measured at 450 nm.

### 4.11. Th17 Cell Differentiation

Naïve CD4^+^ T cells were isolated from mouse splenocytes using magnetic beads (Naïve CD4^+^ T Cell Isolation Kit, Miltenyi Biotec, Bergisch Gladbach, Germany). These cells were then placed in Th cell differentiation media in 24-well plates coated with anti-mouse CD3 and CD28 antibodies and cultured for three days. The Th17 differentiation medium included rmIL-6, hTGF-β, anti-IFN-γ, and anti-IL-4. On the third day, fresh Th cell differentiation media were added, and the cells were cultured for an additional three days. On day six, the cells were collected 4–6 h after being treated with Golgi inhibitors and restimulated with anti-CD3, and Th cell differentiation was analyzed using flow cytometry. CID16020046 at concentrations of 3 and 10 µM was added to the Th differentiation media to evaluate its effect.

### 4.12. Verification of Normality and Statistical Analysis

The results from the animal experiments are expressed as mean ± standard error of the mean (SEM), derived from eight measurements. The Kolmogorov–Smirnov (KS) test was used to assess whether the data followed a normal distribution. Statistical significance was determined using analysis of variance (ANOVA), followed by Tukey’s multiple comparison test, with a significance threshold set at a *p*-value < 0.05. Both the normality assessment and statistical analyses were performed using GraphPad Prism software (version 10.3.1, GraphPad Software, Inc., La Jolla, CA, USA).

## 5. Conclusions

In conclusion, inhibiting GPR55 alleviates the symptoms of collagen-induced rheumatoid arthritis, indicating that GPR55 may serve as a promising therapeutic target for autoimmune diseases by preventing its activation.

## Data Availability

Data are available from the authors upon reasonable request.

## Supplementary Materials

The following supporting information can be downloaded at: https://www.mdpi.com/article/10.3390/ijms26104680/s1.
