# Supplementary material for: GPR55 Antagonist CID16020046 Suppresses Collagen-Induced Rheumatoid Arthritis by Suppressing Th1/Th17 Cells in Mice
Source: Int J Mol Sci. 2025 May 14;26(10):4680. doi: 10.3390/ijms26104680 (PMC12112631; doi:10.3390/ijms26104680)
Supplement: Supplementary file 1 [file ijms-26-04680-s001.zip › ijms-3530271-supplementary.pdf]

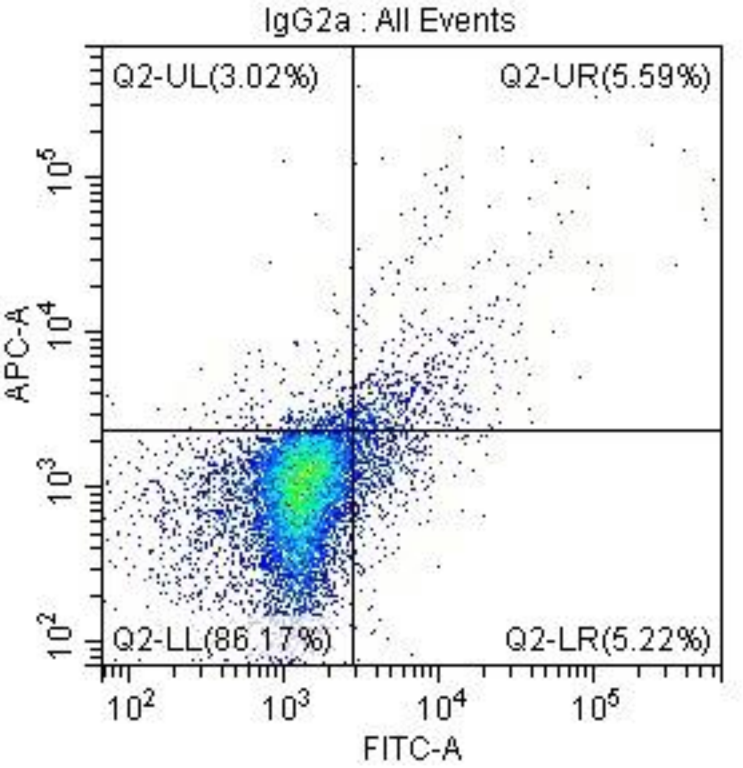

Supplemental Figure S1. FACS results of isotype antibodies for IgG2a labelled with APC-A and FITC-A. Based on the results, we set the thresholds for RORγT, FOXP3, and T-bet.
